# Classification of follicular lymphoma: the effect of computer aid on pathologists grading

**DOI:** 10.1186/s12911-015-0235-6

**Published:** 2015-12-30

**Authors:** Mohammad Faizal Ahmad Fauzi, Michael Pennell, Berkman Sahiner, Weijie Chen, Arwa Shana’ah, Jessica Hemminger, Alejandro Gru, Habibe Kurt, Michael Losos, Amy Joehlin-Price, Christina Kavran, Stephen M. Smith, Nicholas Nowacki, Sharmeen Mansor, Gerard Lozanski, Metin N. Gurcan

**Affiliations:** Faculty of Engineering, Multimedia University, 63100 Cyberjaya, Selangor Malaysia; Division of Biostatistics, College of Public Health, The Ohio State University, Columbus, OH USA; Center for Devices and Radiological Health, US Food and Drug Administration, Silver Spring, MD USA; Department of Pathology, The Ohio State University, Columbus, OH USA; Department of Biomedical Informatics, The Ohio State University, 250 Lincoln Tower, 1800 Cannon Drive, Columbus, OH 43210 USA

**Keywords:** Follicular lymphoma grading, HPF detection, HPF classification, Digital pathology

## Abstract

**Background:**

Follicular lymphoma (FL) is one of the most common lymphoid malignancies in the western world. FL cases are stratified into three histological grades based on the average centroblast count per high power field (HPF). The centroblast count is performed manually by the pathologist using an optical microscope and hematoxylin and eosin (H&E) stained tissue section. Although this is the current clinical practice, it suffers from high inter- and intra-observer variability and is vulnerable to sampling bias.

**Methods:**

In this paper, we present a system, called Follicular Lymphoma Grading System (FLAGS), to assist the pathologist in grading FL cases. We also assess the effect of FLAGS on accuracy of expert and inexperienced readers. FLAGS automatically identifies possible HPFs for examination by analyzing H&E and CD20 stains, before classifying them into low or high risk categories. The pathologist is first asked to review the slides according to the current routine clinical practice, before being presented with FLAGS classification via color-coded map. The accuracy of the readers with and without FLAGS assistance is measured.

**Results:**

FLAGS was used by four experts (board-certified hematopathologists) and seven pathology residents on 20 FL slides. Access to FLAGS improved overall reader accuracy with the biggest improvement seen among residents. An average AUC value of 0.75 was observed which generally indicates “acceptable” diagnostic performance.

**Conclusions:**

The results of this study show that FLAGS can be useful in increasing the pathologists’ accuracy in grading the tissue. To the best of our knowledge, this study measure, for the first time, the effect of computerized image analysis on pathologists’ grading of follicular lymphoma. When fully developed, such systems have the potential to reduce sampling bias by examining an increased proportion of HPFs within follicle regions, as well as to reduce inter- and intra-reader variability.

**Electronic supplementary material:**

The online version of this article (doi:10.1186/s12911-015-0235-6) contains supplementary material, which is available to authorized users.

## Background

Follicular lymphoma (FL) is a cancer of lymph system, and is the second most common lymphoid malignancy in the western world. It is a mature B lymphocyte malignancy of follicular center cell origin. Its diagnosis is based on specific morphologic, immunophenotypic and cytogenetic findings in lymph node/tissue biopsy specimens. Currently the most commonly used risk stratification method is the histological grading (HG) system adopted by the World Health Organization [1]. The HG method requires calculating average count of large malignant cells called centroblasts (CB) per standard microscopic high power field (HPF), equivalent to 0.159 mm^2^. The average CB count per HPF is based on ten randomly selected high power fields within the tissue. The average count stratifies follicular lymphoma cases into three histological grades: Grade I (0–5 CB/HPF), Grade II (6–15 CB/HPF) and Grade III (>15 CB/HPF). Grades I and II are considered a low-risk category while grade III is considered a high-risk category.

The histological grading system, however, suffers from several drawbacks. Firstly, the CB count is performed manually by the pathologist using an optical microscope on hematoxylin and eosin (H&E) stained tissue sections, which leads to subjectivity. It is well documented that the grading system is subject to high inter- and intra-observer variability [2, 3] even among experts [4]. Secondly, since this method uses only ten high power fields, the results are vulnerable to sampling bias. Our group has been developing methods to assist pathologists in reducing inter- and intra-reader variability and sampling bias [5–7]. In this paper, we present a system to assist the pathologist in finalizing the grading of a FL tissue under examination. Given a FL tissue at 40x magnification, the system will automatically identify possible high power fields for examination and classify these HPFs into low (i.g. Grades I or II) or high risk (Grade III) category.

From our observation, there could be between 250 to 1650 high power fields (approximately 2165 × 1365 pixels) that can fit into the tissue section of the FL images. Out of these, 100 to 800 fields may be useful for examination (i.e. within the follicle area). Compared to the ten randomly selected HPFs that are used for grading a typical follicular lymphoma slide, this represents a vast amount of unused data. While it is unrealistic to expect the pathologists to examine such a large number of HPFs, a computer system can do this efficiently and consistently. The proposed system will display all the detected HPFs within the tissue alongside its class (low or high risk). The pathologists can then use this as a second opinion to finalize their grading from the ten randomly sampled HPFs in the HG system. It is important to stress that the system is not intended to replace the pathologists in grading, but rather to provide a second opinion for them to improve their accuracy by reducing the sampling bias. While our previous work documented the positive effect of automatically choosing the HPFs on inter-reader variability, to the best of our knowledge, this is the first work in documenting the effect of computer-aided classification to pathologist grading.

The rest of this paper is organized as follows: Sections 2 explains the image analysis strategies and methods used in detecting the HPFs and classifying them into low and high risk categories, followed by description of the FLAGS system. Section 3 discusses the effect of the proposed system on the expert and inexperienced pathologists involved in the project. Finally, the last section concludes the paper with a summary of the work as well as future directions.

## Methods

### High power fields detection

When pathologists are presented with an FL H&E case, they must select ten HPFs and analyze these HPFs to determine the grade. Selection of the HPFs are left to the pathologists who typically select these regions in areas of follicles while trying to capture as much of the heterogeneity as possible. Considering that there are thousands of potentials HPFs in a given slide, this is not an easy task. Due to the limited time they can allocate reviewing each case, they are limited to these ten HPFs for their decision making.

In most cases, follicles in a FL case are easy to distinguish from the other structures in a slide. However, our previous studies demonstrated that it is easier for the computer algorithms to detect the follicles in immunohistochemically (IHC) stained slides [8–10]. Therefore, we propose a simple thresholding approach to detect suitable high power fields for examination based on CD20-stained images. Compared to H&E images, CD20-stained images provide much better follicle delineation, hence HPFs can be detected better. Our CD20 and H&E biopsy slides were scanned at 40× magnification using a high-resolution whole slide scanner Aperio (Vista, CA) ScanScope™ at the resolution of 0.23 micrometer/pixel. The digitized images have resolution of around 80,000 × 80,000 pixels on average. The cases were selected from the archives of The Ohio State University with Institutional Review Board (IRB) approval (Protocol 2007 C0069, renewed May 7, 2014).

**Fig. 1:**
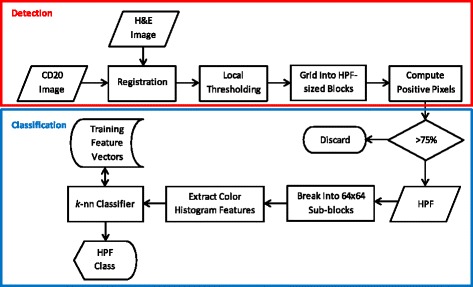
Flowchart of the proposed high power fields detection and classification

Figure 1 (top) shows the flow chart of the proposed detection method. Given the CD20 and H&E stained images of a tissue sample, image registration is carried out to align the tissue boundaries as well as the follicle regions between the two images [11]. Since the classification of the FL tissues will eventually be carried out based on the H&E-stained images, the CD20 images are registered to the H&E images. In other words, the CD20 images are transformed so that they are spatially registered with the H&E images. The saturation channel from the HSV color model is used to register the two images as it provides a good gray level separation between the follicle regions, non-follicle regions, and the white background.

A local thresholding operation is carried out on the registered CD20 images to separate the tissue samples from the background as well as the follicle regions from the non-follicle regions. The local regions were selected to be of size 400 × 400 pixels within HPF blocks of size 1365 × 2165 pixels at 40× magnification. The mean saturation value within each local block is calculated and compared to the threshold value. The threshold value is determined as the value for which the histogram has the peak. In the absence of a peak in the histogram, the default value of 0.5 is used. The output of the local thresholding is a binary image in which the potential follicle regions are marked as positive (i.e. 1). The binary image is then divided into non-overlapping blocks of 1365 × 2165 pixels, and the blocks with more than 75 % positive pixels are deemed suitable high power fields for examination. The choice of 75 % as the threshold was selected after consultation with pathologists, who recommended that at least three quarters of the HPF be within the follicle. Experimental results on 20 randomly selected FL test cases (10 high risks and 10 low risks) suggest that 75 % is a good threshold choice.

Additional file 1: Figure S1 shows a sample image (1a) as it goes through each step of the algorithm (1b-1 g) with the output of the algorithm superimposed on the final image (1 h). It can be observed that CD20 images (both the original and the S channel) provide better follicle delineation (i.e. boundaries of the follicles have higher contrast with the surrounding tissue), and the detected high power fields are all within the follicles. It is important to note that to reduce the computational workload, HPFs can be detected on lower resolution images without sacrificing detection accuracy as the detection is based on the larger follicle regions instead of the individual cells. In our experiment, we use 0.5× magnification, where the tissue slides are reduced to a region of around 1000 × 1000 pixels, high power fields are reduced to a size of 17 × 27 pixels, and the local window size for thresholding is set to a 5 × 5 pixels. The design decisions are made with the understanding that this system has higher tolerance for missed potential HPF regions than incorrect HPF detections. Because pathologists review only 10 HPFs in their current practice, even when the system detects and analyzes 20 high quality HPF regions, this immediately doubles the number of regions reviewed.

### High power field classification

Most of the previous work in the classification of follicular lymphoma was based on the segmentation and recognition of the centroblasts [12–19]. In this work, we propose a simpler alternative by analyzing the centroblasts’ features at the image level. To train the system, 200 high power field images (obtained from different FL cases to the 20 test cases above) were used; for each of these images, at least two pathologists marked every centroblast in these images. A total of 3771 centroblasts were identified from the 200 images and each CB was extracted as a 64 × 64 block for feature extraction purposes. To represent non-centroblast cells, 4000 64 × 64 blocks were then randomly extracted from the non-CB areas of training images (20 from each image). 64-dimensional color histogram [20] features were then extracted from these 3771 centroblast and 4000 non-centroblast blocks which were used to train the system in classifying the detected HPFs from the previous section. We relied on our extensive experience in this area to select the training data set so that the computer can be trained to recognize centroblasts as accurately as possible from all non-centroblast cells regardless of cell type. Most of the 3771 identified centroblasts were in consensus between the pathologists. The 4000 non-centroblasts blocks, on the other hand, were carefully selected such that they are at least twice the distance from any marked centroblast, even those non-consensus ones.

Figure 1 (bottom) shows the flowchart of the proposed high power fields classification method. The detected high power fields are broken into 64 × 64 sub-blocks. For the HPF resolution of 1365x2165 pixels used in our experiment, this resulted in 693 blocks for examination. After color histogram features are extracted from each of these blocks, the *k*-nearest neighbor classification is carried out based on the 3771 centroblast and 4000 non-centroblast training blocks. A block is then classified according to the majority class of its *k* neighbors. An HPF is classified as a *high centroblast field* if its number of sub-blocks classified as centroblasts exceeds a certain threshold *p*. Experimentally, the best classification was achieved when *k* = 49, and *p* = 115. At the tissue level, the tissue will be classified as high risks (grade III) if the number of detected HPFs classified as high exceeds 50 %. Table 1 summarizes the rule-based classification at each level of the process.

**Table 1:** Rule-based classifications at different levels of the process

Levels	Classification Rule
64x64 blocks	Classified as centroblasts region if 50 % of its nearest 49 neighbors are centroblasts
High power fields	Classified as high centroblasts region if the number of 64x64 blocks classified as centroblasts exceeds 115 (out of 693 blocks)
Tissue	Classified as high if 50 % of its detected HPFs are classified as high

Experiments on the 20 FL test cases gives an accuracy of 80 % compared against consensus ground truth. Table 2 summarizes the results.

**Table 2:** Detection and Classification Results for 20 Cases

Case	Grade (Consensus)	Detected HPFs	HPFs Classified as High	HPFs Classified as Low	Percentage of Correct Classification	Grade (Computer)
1	High	826	755	71	91.4	High
2	High	324	320	4	98.8	High
3	High	98	98	0	100.0	High
4	High	56	55	1	98.2	High
5	High	66	66	0	100.0	High
6	High	154	154	0	100.0	High
7	High	208	207	1	99.5	High
8	High	123	120	3	97.6	High
9	High	742	330	412	44.5	Low
10	High	102	6	96	5.9	Low
11	Low	361	273	88	75.6	High
12	Low	187	186	1	99.5	High
13	Low	67	17	50	25.4	Low
14	Low	390	99	291	25.4	Low
15	Low	199	82	117	41.2	Low
16	Low	46	1	45	2.2	Low
17	Low	25	2	23	8.0	Low
18	Low	191	27	164	14.1	Low
19	Low	169	0	169	0.0	Low
20	Low	36	0	36	0.0	Low

### Follicular lymphoma grading system (FLAGS)

All 20 cases were used in a study of the influence of the proposed computer detection and classification of FL tissues on pathologist accuracy. As shown in Table 1, using a threshold of 50 % for computer scores, sixteen of these cases were correctly classified by the computer system, and four were incorrectly classified. The four incorrectly classified cases were included to see if the pathologists can still trust the computer even after they encounter such cases. The pathologists however, were not informed about the classification accuracy and cases were presented to them in a random order.

For each case, the ground truth was generated by extensive reading by two experienced pathologists, where each and every centroblast in the selected HPFs was identified and counted. Eleven pathologists (four experts and seven residents) were involved in the experiments, and each pathologist was asked to grade twenty cases of H&E stained follicular lymphoma slides. The confidence scores were then used to perform Receiver Operating Characteristic (ROC) analyses comparing reader accuracy before and after using the computer.

Additional file 2: Figure S2 shows the user interface of our proposed system designed to measure the influence of the computer grading of FL tissues on the pathologists. The system is fairly easy to use thus brief step-by-step training was given to each pathologist before they proceeded to grade the 20 FL cases. Additional file 3: Figure S3 shows an example of the HPF classification map generated by the system, where red boxes indicate low CB regions, while green boxes indicate high CB regions. The detailed usage process of the system is described below:

A: Conventional reading:
  1. Pathologist selects a case to be examined. For this phase of the review, the pathologist is instructed to review the case in a standard clinical setting; i.e., using glass slides and under the microscope.
  2. Pathologist will review the case under a microscope to count the number of centroblasts in ten randomly selected high power fields, as in standard clinical practice.
  3. The counts are recorded into the computer using the interface on the left hand panel of Additional file 2: Figure S2. It should be noted that this is just for convenience (as opposed to writing the counts on a piece of paper) and no digital reading or assistance is provided at this stage.
  4. Once the ten counts have been entered, the system will show the grade based on World Health Organization (WHO) guidelines. Again, this is for convenience for the pathologists, the computer simply calculates the average count so that they do not need to use a calculator to carry out this step. Up to this point, the pathologist carries out the reading in the conventional way.
B: Computer-assist:
  5. The computer will ask the pathologist to provide a score in the range: 0–100, reflecting how confident they are that the case is high risk (Grade III).
  6. For the whole tissue sample, the computer will identify suitable high power fields and classify them into low (grade I and II) or high (grade III) category regions. The pathologist can view and verify these high power fields by clicking on the colored boxes (see Additional file 3: Figure S3).
C: Decision:
  7. The system will then ask the pathologist if he or she wants to change their confidence score after seeing the computer’s output.

The pathologists were asked to follow the standard practice in follicular lymphoma grading; i.e. to look at 10 random high power fields within the tissue, and to count the number of centroblasts in each of the 10 locations. The average number of centroblasts per high power field was calculated and a case was marked as high grade if the average number was more than 15 in accordance with the current WHO guidelines. The only difference between the current clinical practice and our set up is that the computer calculates the averages instead of pathologist using a calculator to compute the average value. After the average centroblast counting, the system proceeds to ask “In your opinion, how likely is this Grade III?” Then, the pathologists are prompted to enter a score in the range of 0 (unlikely) to 100 (very likely).

The second part of the system shows the pathologists the computer detected HPFs as well as classification for each HPF according to the classifier explained in Section 3. The classification was presented in color code so that high grade HPF regions were marked in green outline and low grade regions in red. To understand the computer’s classification decisions, the pathologists were given the option to click on any of the detected high power field regions to view the area at high resolution (i.e. 40x). After showing the classification, the system proceeded to ask “After seeing the computer’s output, how likely is this Grade III?” To answer this question, the pathologists have the options to maintain or change their previously entered likelihood score. All the pathologists’ ten CB counts, average CB count, initial likelihood score and final likelihood score were recorded for analysis.

It is important to point out that, as this study was intended as a proof of concept, our readers had to go through extra steps to enable our extensive statistical analysis. Once these studies are completed and the system is redesigned to be deployed clinically, several of the steps in the current system (e.g. recording confidence interval before and after reviewing the computer results) will not be necessary and the whole process will be very streamlined. The FLAGS system was designed to guide pathologists towards the most informative areas on the slide. The final classification decision is that of the pathologist. Pathologists with experience in FL diagnosis have a choice of agreeing or disagreeing with computer marking of centroblasts by re-reviewing the areas of interest highlighted by the computer as centroblast rich.

Since pathologists will likely review only a small number of HPFs with computer aid, the additional time is kept to a minimum. Once the computer identifies the HPFs, it is up to the pathologist to select which computer-classified HPFs they want to inspect more closely. We preferred this approach because it gives more flexibility to the pathologist in terms of whether they prefer computer assistance, and if they do, the extent of the assistance. This is a more realistic scenario as in regards to how such a system might be used in practice, as opposed to pre-specifying which HPFs the pathologist should inspect more closely with the computer aid.

## Results and discussion

Two pathologists were considered to agree on a case if they both rated the case as high grade (score > =50), or if they both rated the case as low grade (score < 50). It is important to point out that from the experiment, even expert pathologists disagree on the grading for some of the cases. Out of the 20 cases, only nine cases were unanimously agreed upon by the expert pathologists. The interrater agreement improved to ten cases upon seeing the computers’ output. Thus, the agreement—when taking into account resident pathologists reading as well—is worse: only a single case registered unanimous agreement, which improved to four cases upon seeing the computer’s output. Additional file 4: Figure S4 plot the change in median confidence scores (initial vs. final scores) across 11 pathologists for the 20 cases.

### Agreement with the ground truth and computer output

We used a threshold of 50 on pathologists’ scores to define agreement with ground truth. Thus, if the ground truth for a case was high risk, a pathologist was considered to be in agreement with the ground truth if his/her score was equal to or larger than 50. If the ground truth for a case was low risk, a pathologist was considered to be in agreement with the ground truth if his/her score was less than 50. Overall, out of 220 readings (20 cases × 11 pathologists), only 125 initial readings were in agreement with the ground truth (56.8 %). Agreement improved to 148 (67.3 %) after the pathologists reviewed the computer’s output.

#### Analysis according to pathologists’ experience level

Comparing expert pathologists to residents, 53.8 % (43 out of 80) of the experts’ readings were initially in agreement with the ground truth, while 58.6 % (82 out of 140) of the residents’ readings were initially in agreement. These numbers improved to 62.5 % and 70.0 %, respectively upon reviewing the computer’s output. While the initial reading was more or less similar between the experts and inexperienced readers, it is interesting to note that residents were more inclined to change their score after seeing the computer results.

We analyzed the agreement data using the Obuchowski-Rockett (OR) method [21] with Hillis denominator degrees of freedom [22] to estimate confidence intervals and to compare the agreement with and without the use of the computer system. Although the OR method is typically used to analyze ROC data, it is equally applicable to other indexes of diagnostic accuracy, including agreement [23]. Table 3 summarizes the agreement results for the group of 11 pathologists, as well as for the expert pathologist and resident subgroups.

**Table 3:** Percent agreement with ground truth and Average Area Under the ROC Curve (AUC) Estimates

	Without Computer		With Computer		
	Agreement (%)	95 % CI	Agreement (%)	95 % CI	*p*-value of the difference
Experts	53.8	(37.2, 70.3)	62.5	(47.3, 77.7)	0.188
Residents	58.6	(44.0, 73.1)	70.0 (56.9, 83.1)	0.014	
All Readers	56.8	(42.3, 71.1)	67.3	(53.6, 80.1)	0.004
	AUC	95 % CI	AUC	95 % CI	*p*-value
Experts	0.62	(0.40, 0.84)	0.69	(0.50, 0.87)	0.21
Residents	0.66	(0.46, 0.86)	0.79	(0.63, 0.95)	0.04
All Readers	0.65	(0.46, 0.83)	0.75	(0.58, 0.92)	0.03

Another analysis that can be made is on the agreement to the computer’s output, instead of to the ground truth. Overall 153 initial readings (69.6 %) are in agreement with the computer classification, which increases to 176 readings (80.0 %) after consulting the computer output. From these numbers, the experts registered an initial 68.8 % agreement which later improved to 77.5 %, while the residents recorded an initial 70.0 % agreement that subsequently improved to 81.4 %. As in the comparison against the ground truth, the agreement increases after seeing the computer’s output, and the resident pathologists are more willing to alter their confidence score.

In terms of overall changes in the agreement between the pathologists and the ground truth, 25 out of the 220 readings (8 from the experts and 17 from the residents) recorded a change towards computer results, with only two cases (one from an expert and one from a resident) recorded the change in the opposite direction. This indicates that although the pathologists were not informed about the performance level of the computer system, both experts and residents developed a trust for the computer system for their interpretation.

#### Analysis according to correctly and incorrectly classified cases by the computer system

We used a threshold of 50 on the computer scores to define correctly and incorrectly classified cases by the computer system. Thus, if the ground truth for a case was high risk, it was considered to be correctly classified if the computer score was equal to or larger than 50. The 220 readings can be subclassified into 88 readings of correctly classified high grade cases (8 high grade cases × 11 pathologists), 88 readings of correctly classified low grade cases (8 low grade cases × 11 pathologists), and 44 readings of incorrectly classified cases (4 incorrectly classified cases × 11 pathologists). It was observed that the pathologists performed better in grading the correctly classified high grade cases compared to the correctly classified low grade cases, for both initial and final grading. Before computer results were presented, 69 out of the 88 correctly classified high grade readings (78.4 %) were in agreement with the ground truth, while only 48 of the 88 low grade readings (54.6 %) were. These numbers improved to 78 and 62 cases respectively (88.6 % and 70.5 %) after consulting the computer results. Contrarily, only 8 of the 44 initial readings of incorrectly classified cases (18.2 %) were in agreement with the ground truth, which remained the same for the final grading.

The agreement against the computer‘s output shows more or less the same improvement between initial and final confidence scores as in the agreement against the ground truth. In terms of actual confidence scores, for correctly classified high grade cases, the average final confidence score was 82.2 % compared to 73.1 % initially. For the correctly classified low grade cases, the average final confidence score was 28.8 % compared to 43.1 % initially. As expected, the average scores for the incorrectly classified cases approximated the 50 % mark (46.8 % initial, 53.9 % final).

### ROC analysis

**Fig. 2:**
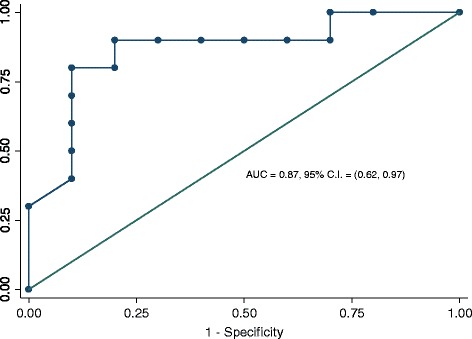
ROC Curve for Stand-alone Computer Diagnosis of Grade III

Stand-alone computer ratings of the likelihood that a case was Grade III (0–100 scale) were compared to the ground truth by calculating the Area Under the ROC Curve (AUC) using the trapezoidal rule and, by viewing the AUC as a binomial proportion [24], an exact binomial 95 % confidence interval of the trapezoidal AUC was obtained. The confidence interval was also calculated using U-statistics to estimate the variance of AUC [25], followed by the logistic transform to find the confidence interval for logit (AUC), and transforming the confidence interval back to AUC [26]. The two methods resulted in similar 95 % confidence intervals. ROC curves of the computer and individual readers were generated using Intercooled Stata 11 (StataCorp, College Station, TX). The computer exhibited excellent performance in discriminating high grade from low grade cases, as indicated by the ROC curve shown in Fig. 2.

The Obuchowski-Rockett (OR) method [21] with Hillis denominator degrees of freedom [22] was used to compare the average AUC of readers with and without the computer. To determine if the effects of the computer differed by experience level, we also performed separate OR analyses for expert hematopathologists and residents. All AUC calculations were made using the nonparametric trapezoidal method for integration. The OR analysis was performed in SAS Version 9.2 (SAS Inc., Cary, NC) using the MRMC_ANALYSIS macro by Hillis et al. [27]. The average ROC curves corresponding to the average AUC values were generated using a nonparametric averaging method described in [28].

**Fig. 3:**
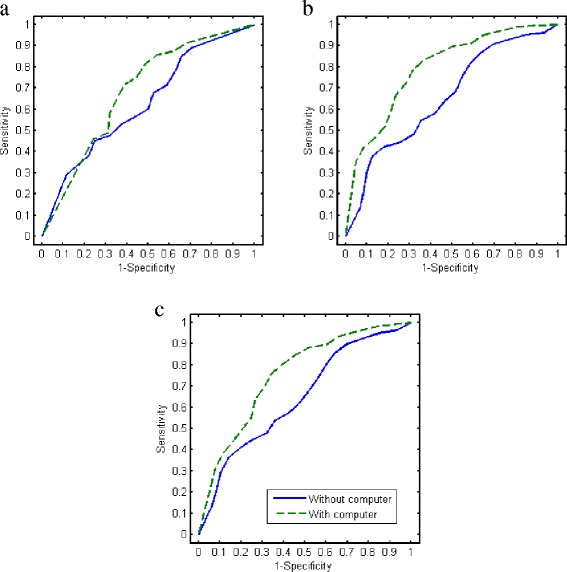
Average ROC curves obtained using a nonparametric average [26] of empirical ROC curves of (**a**) the four expert readers, (**b**) the seven resident readers, and (**c**) all 11 readers. See Table 3 for the corresponding average AUC values and statistical inference results

Additional file 5: Figure S5 shows the ROC curves for each and every individual reader. Table 3 shows the results for statistical comparisons of the mean AUC performance without the computer aid versus with the computer aid for different reader populations. Figure 3 shows the average ROC curves corresponding to those average AUC values in Table 3, where the average was performed along the direction of the diagonal line connecting the upper-left point and the lower-right point in the ROC space. These results indicate that access to the computer improved reader accuracy with the biggest improvement seen among residents. However, even with the improvements provided by computer, the average AUC value was only 0.75, which is generally regarded as “fair” or “acceptable” diagnostic performance (see, for example, [29]).

Overall, the convergence of the final grading towards the ground truth and computer results, as well as the improvement of the confidence scores (towards 100 % for high risk cases and towards 0 % for low risk cases) suggests that with proper image analysis methods that can yield acceptable accuracy, the computer detection and classification does help in increasing the confidence of the pathologists in their grading ability.

## Conclusions

We have proposed a system to assist pathologists in grading follicular lymphoma cases. The system first identifies potential high power fields for examination by analyzing accompanying CD20 images. Even at relatively small false positive rates, the number of computer selected high power fields is much higher than 10 (i.e. the number of randomly selected high power fields in the current clinical practice) and represents only a tiny fraction of all available high power fields in a given slide. These high power fields are then classified into low or high grades based on the *k*-nearest neighbor classifier of potential centroblast regions within sub-blocks of the HPF regions followed by rule-based classification at the block, HPF and tissue levels. The proposed classification methods are able to achieve an accuracy of 80 % for this data set. The detected and classified HPFs are then presented as an HPF classification map for pathologists’ review and verification. To the best of our knowledge, for the first time, we measure the effect of computer-detection on the decisions of experts and inexperienced readers in grading follicular lymphoma. The average AUC value of the 11 readers increased from 0.65 to 0.75 when they used the computer system, which is generally considered acceptable diagnostic performance. Residents were found more likely to change their scores after using the computer which could explain why their readings were more accurate than the pathologist readings.

The current study is performed on set of cases with classical follicular pattern that is predominant in FL clinical cases, thus non-follicular patterns in follicular lymphoma may pose problem for our system as it is deployed right now. Recognizing the important issue of FL with diffuse pattern and fused follicles, we have been working on an algorithmic solution that will recognize characteristic staining for follicular dendric cells networks (FDCS) (as stained by CD21, CD23 or CD35 IHC stains) to help with this problem. We believe that digital computer triangulation of CD20 and FDCS positive areas will accurately guide our algorithm towards proper diagnostic areas in FL cases with diffuse pattern and with fused follicles.

Finally, due to the limited number of case samples available to us at the time of the study, some parameters of the computer system were selected based on the regions of interest extracted from the twenty cases that were used in the study with the pathologists. This may have optimistically biased the performance of the computer system, which had an AUC value of 0.87, higher than any of the pathologists who participated in the study. In the future, we intend to apply the designed computer system to a completely independent, randomly selected data set of tissue samples, and investigate if the computer performance on the current data set is biased. However, regardless of the potential bias, we demonstrated for the first time that a computer system with the characteristics and performance described in this study has the potential to increase both the percent agreement with the ground truth and the AUC value of the pathologists in the task of follicular lymphoma grading. When fully developed, the proposed system has the potential to reduce sampling bias, thus decreasing potential errors. Our future work will focus on further improving the classification accuracy of both the detection and classification algorithms, as well as expanding the study on a larger number of cases and more pathologists.

## Additional files

Additional file 1: Figure S1. Flow of the detection process: (a) H&E image, (b) CD20 image, (c) S channel of H&E, (d) S channel of CD20, (e) Registered CD20 (S channel), (f) Local thresholding with blocks showing detected HPF regions, (g) Detected HPFs on CD20 (S channel), (h) Detected HPFs on the H&E. (DOCX 384 kb) Additional file 2: Figure S2. Graphical User Interface of the proposed FLAGS system. (DOCX 1052 kb) Additional file 3: Figure S3. Example of the HPF classification map generated by the system (top), and the zoomed version (40x magnification) of one of the detected HPF. (DOCX 3320 kb) Additional file 4: Figure S4. Initial and final median confidence scores across 11 pathologists for the 20 cases. The first eight readings are for correctly classified high grade, the middle four readings are for the incorrectly classified cases, and the last eight readings are for correctly classified low grade. (DOCX 138 kb) Additional file 5: Figure S5. Reader-Specific ROC Curves. Readers 1-4 were expert hematopathologists. Readers 5-11 were residents. (DOCX 21 kb)

## Abbreviations

FL: Follicular lymphoma
HPF: High power field
H&E: Hematoxylin and eosin
FLAGS: Follicular Lymphoma Grading System
WHO: World Health Organization
HG: Histological grading
CB: Centroblasts
IHC: Immunohistochemically
IRB: Institutional review board
ROC: Receiver operating characteristic
AUC: Area under the ROC Curve
OR: Obuchowski-Rockett method
