# Supplementary figures and images for: Classification of follicular lymphoma: the effect of computer aid on pathologists grading
Source: BMC Med Inform Decis Mak. 2015 Dec 30;15:115. doi: 10.1186/s12911-015-0235-6 (PMC4696238; doi:10.1186/s12911-015-0235-6)

**
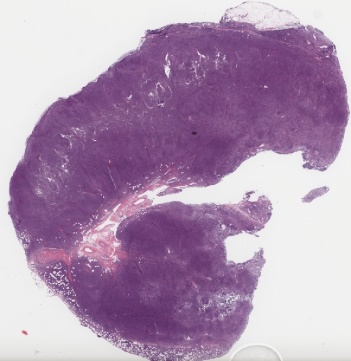

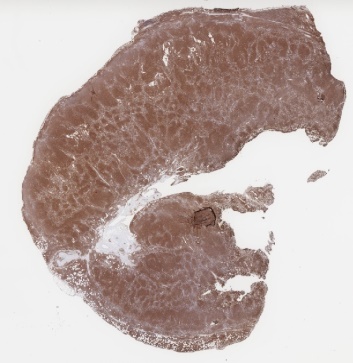

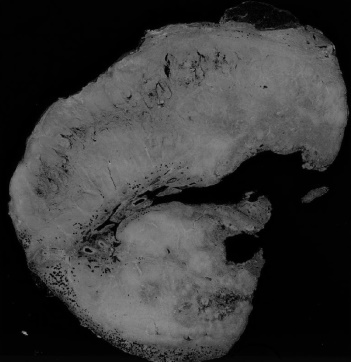

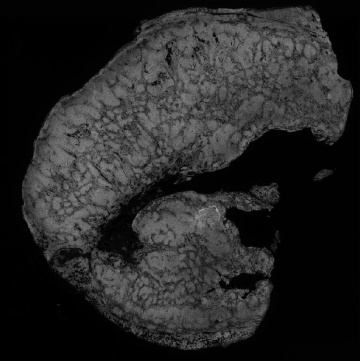
**

(a) (b) (c) (d)

**
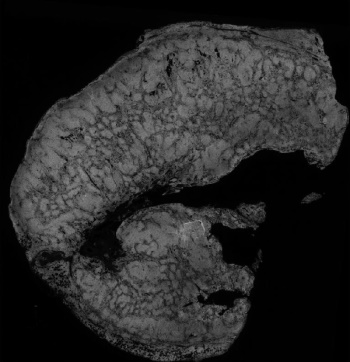

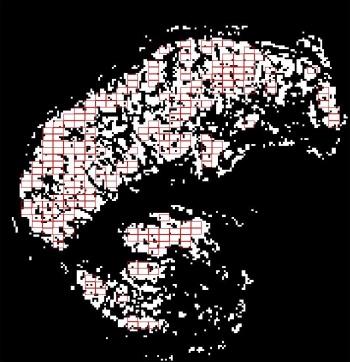

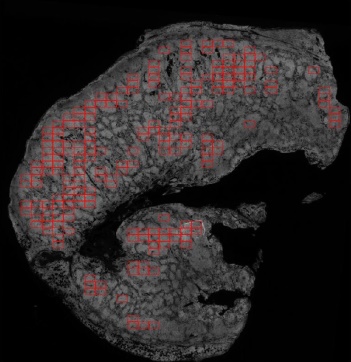

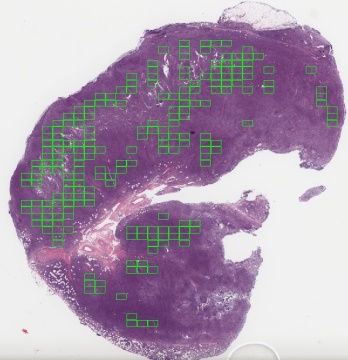
**

(e) (f) (g) (h)

Supplement: Additional file 1: Figure S1. — Flow of the detection process: (a) H&E image, (b) CD20 image, (c) S channel of H&E, (d) S channel of CD20, (e) Registered CD20 (S channel), (f) Local thresholding with blocks showing detected HPF regions, (g) Detected HPFs on CD20 (S channel), (h) Detected HPFs on the H&E. (DOCX 384 kb) [file 12911_2015_235_MOESM1_ESM.docx]

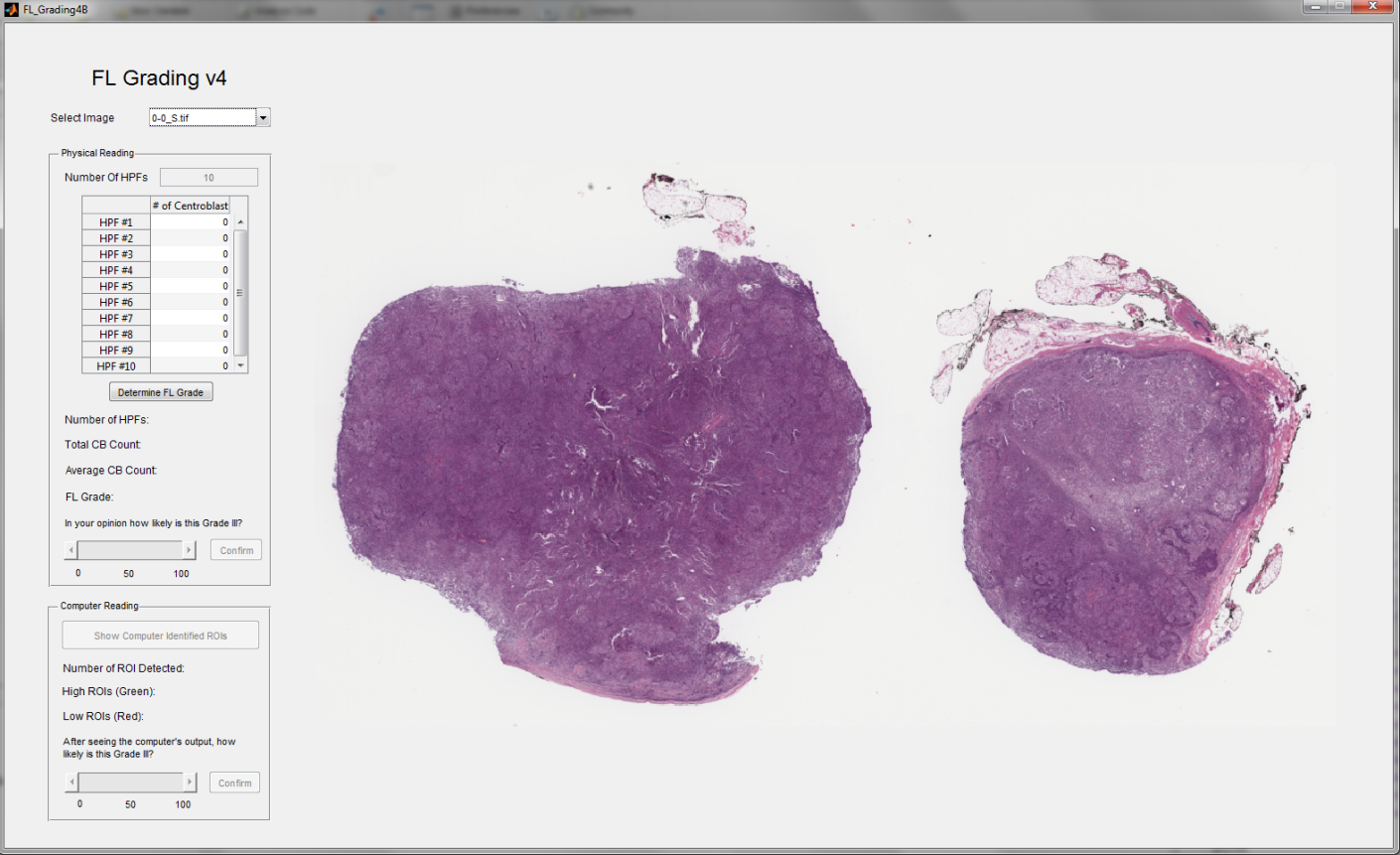

Supplement: Additional file 2: Figure S2. — Graphical User Interface of the proposed FLAGS system. (DOCX 1052 kb) [file 12911_2015_235_MOESM2_ESM.docx]

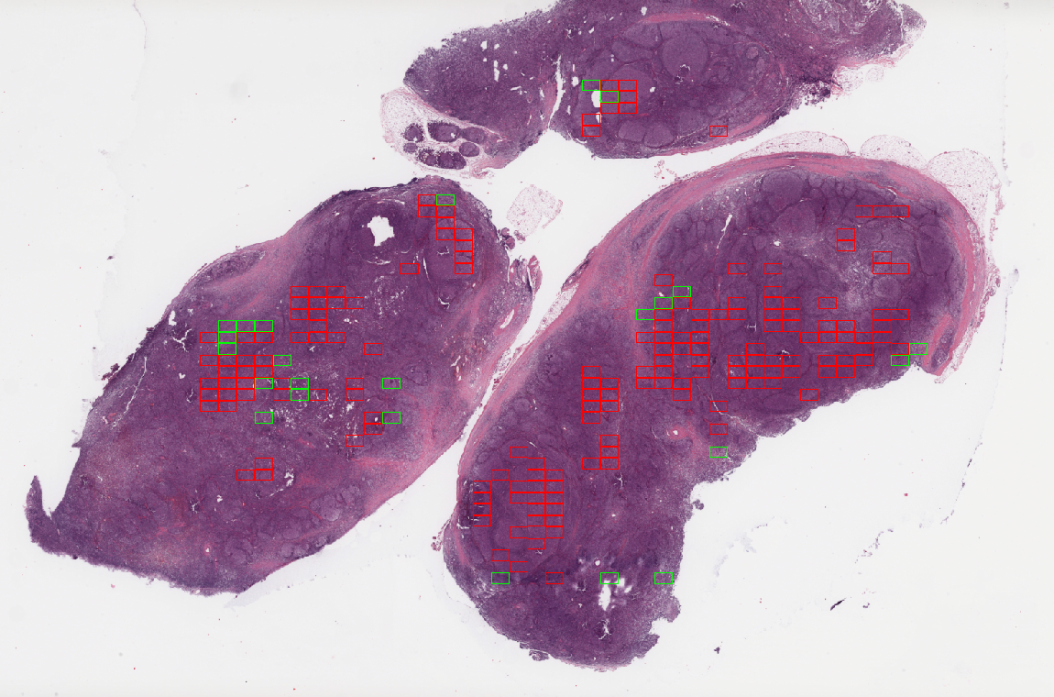


**
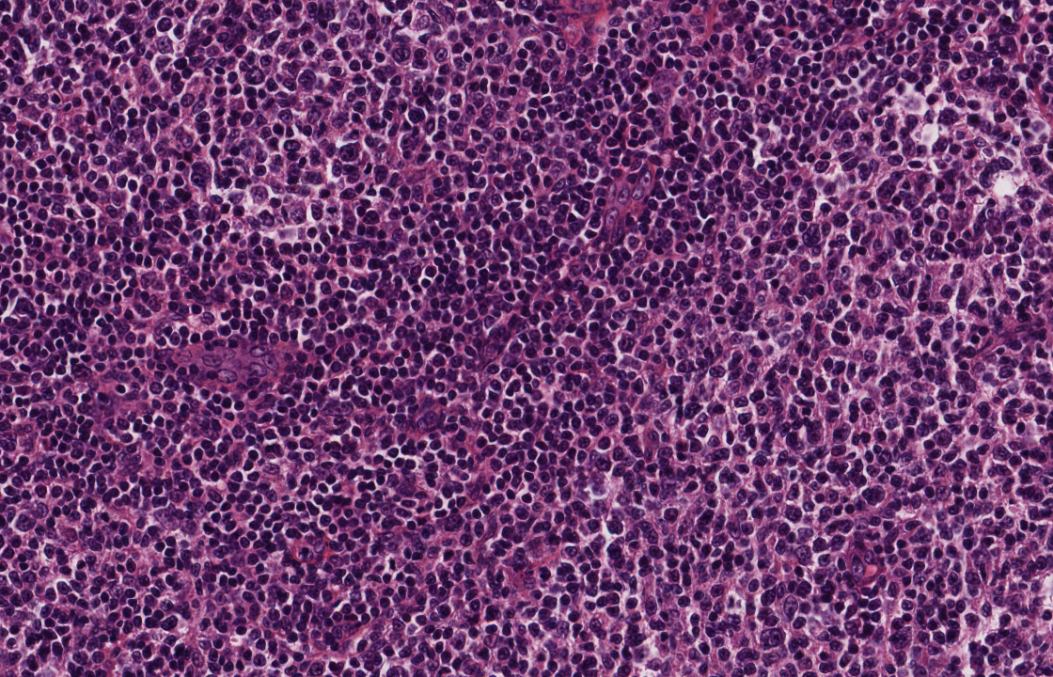
**

Supplement: Additional file 3: Figure S3. — Example of the HPF classification map generated by the system (top), and the zoomed version (40x magnification) of one of the detected HPF. (DOCX 3320 kb) [file 12911_2015_235_MOESM3_ESM.docx]
