# Supplementary material for: Classification of follicular lymphoma: the effect of computer aid on pathologists grading
Source: BMC Med Inform Decis Mak. 2015 Dec 30;15:115. doi: 10.1186/s12911-015-0235-6 (PMC4696238; doi:10.1186/s12911-015-0235-6)
Supplement: Supplementary file 2 [file 12911_2015_235_MOESM4_ESM.docx]

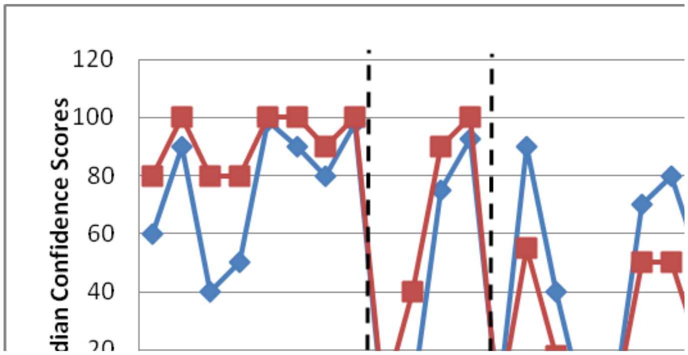

120
100
80
60
40
20
dian Confidence Scores
