# Supplementary material for: Classification of follicular lymphoma: the effect of computer aid on pathologists grading
Source: BMC Med Inform Decis Mak. 2015 Dec 30;15:115. doi: 10.1186/s12911-015-0235-6 (PMC4696238; doi:10.1186/s12911-015-0235-6)
Supplement: Supplementary file 3 [file 12911_2015_235_MOESM5_ESM.docx]

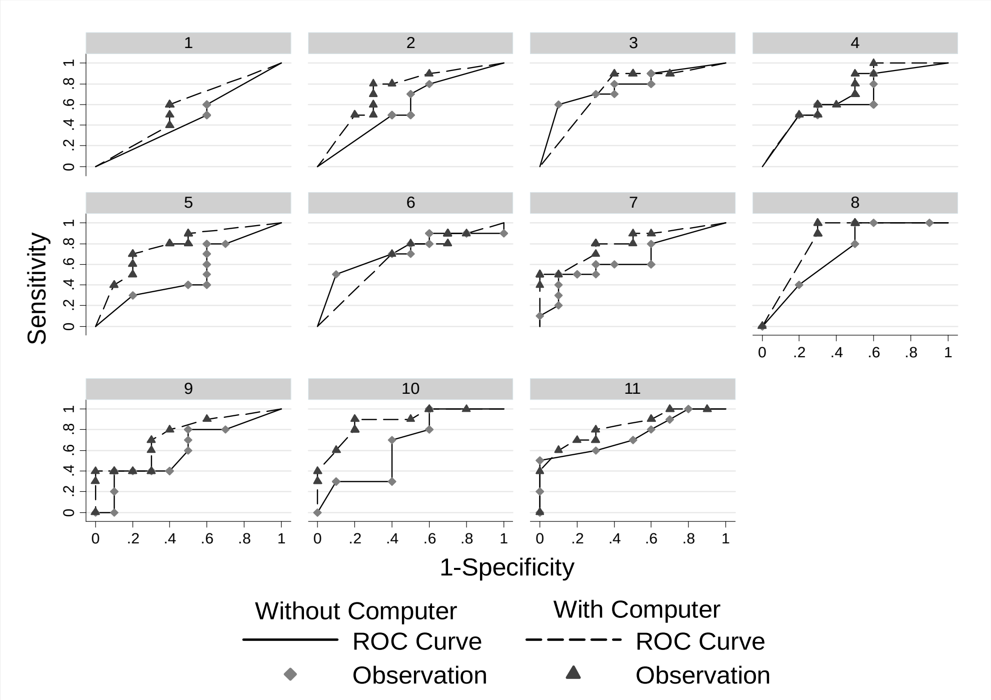

1
2
3
4
5
6
7
8
9
10
11
Sensitivity
1-Specificity
0
.2
.4
.6
.8
1
Without Computer
ROC Curve
Observation
With Computer
ROC Curve
Observation
